# Bayesian Analysis of a Lipid-Based Physiologically Based Toxicokinetic Model for a Mixture of PCBs in Rats

**DOI:** 10.1155/2012/895391

**Published:** 2012-01-19

**Authors:** Alan F. Sasso, Panos G. Georgopoulos, Sastry S. Isukapalli, Kannan Krishnan

**Affiliations:** ^1^Environmental and Occupational Health Sciences Institute, UMDNJ-RW Johnson Medical School, Rutgers University, 170 Frelinghuysen Road, Piscataway, NJ 08854, USA; ^2^Department of Chemical and Biochemical Engineering, Rutgers University, 98 Brett Road, Piscataway, NJ 08854, USA; ^3^Département de Santé Environnementale et Santé au Travail, 2375 Cote-Sainte-Catherine, Université de Montréal, Montreal, Road QC, Canada H3C 3J7

## Abstract

A lipid-based physiologically based toxicokinetic (PBTK) model has been developed for a mixture of six polychlorinated biphenyls (PCBs) in rats. The aim of this study was to apply population Bayesian analysis to a lipid PBTK model, while incorporating an internal exposure-response model linking enzyme induction and metabolic rate. Lipid-based physiologically based toxicokinetic models are a subset of PBTK models that can simulate concentrations of highly lipophilic compounds in tissue lipids, without the need for partition coefficients. A hierarchical treatment of population metabolic parameters and a CYP450 induction model were incorporated into the lipid-based PBTK framework, and Markov-Chain Monte Carlo was applied to *in vivo* data. A mass balance of CYP1A and CYP2B in the liver was necessary to model PCB metabolism at high doses. The linked PBTK/induction model remained on a lipid basis and was capable of modeling PCB concentrations in multiple tissues for all dose levels and dose profiles.

## 1. Introduction

Polychlorinated biphenyls (PCBs) are industrial chemicals that have persisted in the environment despite widespread international bans beginning in the 1970s [1]. There are a total of 209 possible PCB congeners, and many of these co-occur in the environment based on the composition of commercially produced PCB mixtures [2]. Mixtures of PCBs are commonly detected in blood samples of the human population, with estimated elimination half-lives of up to 10–15 years [3]. Assessing risks from these mixtures is complicated by the significant variability of toxicological properties of individual PCBs, the time-varying changes in the composition of PCB mixtures in the environment [4], and the metabolic interactions among individual PCBs in the body [5–7].

Physiologically based toxicokinetic (PBTK) models are well-established tools for simulating internal doses and biomarkers of environmental contaminants [8]. PBTK modeling for mixtures of chemicals has gained prominence for risk assessment applications and provides a means for capturing the various types of metabolic interactions among individual constituents [9, 10]. However, for complex mixtures, PBTK models typically need a large number of parameters and often require significant time and data for model development and evaluation. Approaches that minimize the number of parameters in mixture PBTK models while still capturing the major interactions can help reduce such data burdens.

For the class of highly lipophilic compounds such as PCBs and dioxins, one approach for PBTK model reduction is the use of lipid-based models, which assume contaminants only accumulate in the lipids of tissues and blood [11, 12]. Lipid-based PBTK models do not require tissue/blood partition coefficients, which significantly reduces the number of chemical-specific parameters needed for modeling the toxicokinetics of complex mixtures. In these models, residence times in each compartment are assumed to be dependent on tissue lipid volumes and lipid flow rates, which are chemical-independent. Under such scenarios, chemical-specific parameters are limited to absorption, metabolism, elimination, and metabolic interactions. Lipid-based PBTK modeling provides a generalized treatment of highly lipophilic chemicals, leading to more efficient modeling of complex mixtures (e.g., Emond et al. [11]).

However, parameterization and optimization of lipid-based PBTK models present challenges due to the reduced degrees of freedom, since partition coefficients for each tissue-chemical combination are not considered. This decreased flexibility requires the use of sophisticated parameter estimation techniques for reducing model errors, especially when experimental data include substantial population variability. Bayesian parameter estimation techniques are highly useful in handling such complex population parameter estimation and optimization problems [13]. To date, lipid-based PBTK models for mixtures of chemicals have not been widely used. This study involves the development of a lipid-based PBTK model for a mixture of PCBs, and subsequent model parameterization, refinement, and optimization using Bayesian parameter estimation techniques.

## 2. Methods

### 2.1. Data

The data published by Emond et al. [11] consisted of rats receiving oral doses of a mixture of 6 PCB congeners: 118 (2,3′,4,4′,5-pentachlorobiphenyl), 138 (2,2′,3′,4,4′,5-hexachlorobiphenyl), 153 (2,2′,4,4′,5,5′-hexachlorobiphenyl), 170 (2,2′,3,3′,4,4′,5-heptachlorobiphenyl), 180 (2,2′,3,4,4′,5,5′-heptachlorobiphenyl), and 187 (2,2′,3,4,5,5′,6-heptachlorobiphenyl). The dosing regimen consisted of 3 dose levels (5, 50, and 500 *μ*g/kg body weight of each PCB), and 4 dose protocols (one dose per day, one dose per week, consecutive daily doses for 13 days followed by no exposure, and 13 irregularly timed doses). Rats were either sacrificed at 41 days or 90 days for data collection. PCB concentrations in total lipids of plasma, liver, and adipose tissue were measured (adipose tissue concentrations were measured for only those rats sacrificed at 90 days). Body weight and liver weight at time of sacrifice were measured. The final data consisted of approximately six rats for each dose level/dose protocol*/*sacrifice day combination (142 rats in total) [11].

### 2.2. Toxicokinetic Model

The new PBTK model developed here is an extension of the lipid-based PCB mixture model by Emond et al. [11]. The original model formulation required alternative clearance parameters at different dose levels and dose protocols, thus increasing the number of parameters and creating model discontinuity. The updated model provides an alternative formulation that incorporates CYP450 induction, thus facilitating the use of a single set of parameters for wider applicability of the model.

The model consists of five compartments (blood, adipose tissue, liver, slowly and rapidly perfused), with mean physiology defined in Table 1. The overall clearance of PCBs is empirically described in the liver and represented as a function of CYP450 metabolism (metabolites are subsequently excreted in urine or feces [18]), which is modeled as a first-order process [11]. It is assumed that PCBs accumulate only in the neutral lipid spaces of blood and tissues. Any accumulation outside of the lipid fraction is assumed to be negligible and is not incorporated into the mass balance. Compartment volumes correspond to the lipid volumes in each tissue, and the total cardiac output is corrected for the fractional lipid content of blood. The PBTK model is based on chemical concentration in neutral lipid equivalent (NLE) components of blood and tissues, which can be converted to concentration in total lipids (the measurable quantity [14]). The lipid-based mass balances for tissues in the PBTK model are defined in the same manner as the original model [11]:

(1) $$\begin{array}{c} \frac{dA_{\text{nlt}}}{dt}=Q_{\text{nlt}}\left(C_{\text{nla}}-C_{\text{nlt}}\right),\\ C_{\text{nlt}}=\frac{A_{\text{nlt}}}{V_{\text{nlt}}},\\ C_{\text{tlt}}=C_{\text{nlt}}\frac{V_{\text{nlt}}}{V_{\text{tlt}}}, \end{array}$$

where *A*_nlt_ is the mass of chemical in the tissue NLEs (*μ*g), *Q*_nlt_ is the flow rate of blood NLEs through the tissue (mL NLE/h), *C*_nla_ is the chemical concentration in the NLE fraction of arterial blood (*μ*g/mL NLE), *C*_nlt_ is the chemical concentration in the tissue NLEs (*μ*g/mL NLE), *C*_tlt_ is the chemical concentration expressed in terms of total lipids in tissue (*μ*g/mL of total lipid), *V*_nlt_ is the volume of neutral lipid equivalents in tissue (mL NLE), and *V*_tlt_ is the volume of total lipids in tissue (mL total lipid). Volumes of total lipids in tissues are measurable quantities, while neutral lipid equivalents are quantities that are derived by assuming NLEs are composed of all the neutral lipids and 30% of the phospholipids in tissue [11, 14].

NLE-based volumes in Table 2 are obtained by multiplying conventional values with NLE ratios in Table 1. Flows are obtained by multiplying conventional values with the blood NLE ratio. The ratio of NLE/total lipid in Table 2  (*V*_nlt_/*V*_tlt_) is used to convert concentrations from NLE basis to total lipid basis. To convert liver, fat, and plasma NLE concentrations to a total lipid basis, the corresponding values in Table 1 (column 3) are used.

### 2.3. Induction Model

High chronic doses of the PCB mixture caused an increased elimination rate for all PCBs, which was attributed to CYP450 induction [11]. PBTK models predicting changes in metabolic rate due to CYP450 induction have been previously implemented for other chemicals [17, 20–23]. A CYP450 balance in the liver can be defined as

(2) $$\begin{array}{c} \frac{dA^{\text{CYP}}}{dt}=k_{0}-k_{e}\times A^{\text{CYP}}+S\left(t\right), \end{array}$$

where *A* ^CYP^ is the mass (per gram protein) of CYP450 in the liver, *k*_0_ is the basal CYP450 production rate (mass/time), *k*_*e*_ is the CYP450 degradation rate (time^−1^), and *S*(*t*) is the stimulation function for induction exposure response (mass/time). The initial condition for *A* ^CYP^ is the baseline level *A*_0_^CYP^. In the presence of zero inducer, *S*(*t*) is zero and (2) is at steady state, and, therefore, *k*_0_ is equivalent to *k*_*e*_ *A*_0_^CYP^. 

For simplicity, a linear function was adopted for *S*(*t*). While a Hill equation could have been implemented, it was determined that optimizing Hill parameters with weak prior information was impractical. The internal exposure-response parameters of this particular mixture are highly uncertain. Furthermore, lipid-based model formulations assume that contaminant concentration outside of the lipid space is negligible. If only unbound chemical outside of the lipid space can initiate a toxicological response (i.e., by binding to a receptor), a lipid model will assume that this external/unbound concentration remains low and does not approach saturation. The initiation of CYP450 induction was modeled as proportional to the inducing PCB concentration:

(3) $$\begin{array}{c} S\left(t\right)=k_{0}{FC}_{\text{IND}}, \end{array}$$

where *C*_IND_ is a relative metric for inducer concentration, and *F* (≥0) is the induction slope factor defining the increase in CYP450 enzyme production caused by *C*_IND_. The induction slope is defined as a factor of the basal production rate.

In previous PBTK models for lipophilic contaminants, the inducer concentration *C*_IND_ was defined to be the chemical concentration bound to the Ah-receptor [17]. The Ah-receptor has consistently been demonstrated to be crucial for CYP450 induction by PCBs [24]. Since only unbound chemical outside of the lipid space can bind to the Ah-receptor [23], assumptions from the prior PBPK models do not apply. To maintain the parsimony of the model and maintain lipid-based concentrations throughout, *C*_IND_ was defined as the concentration of the inducing PCB congener(s) in the neutral-lipid space of the liver. Any additional steps in the induction process (i.e., concentration gradients between free and lipid-space PCB, and Ah-receptor binding) were essentially lumped into the dose response parameter *F*. During the model development phase, it was observed that introducing a time-lag into the stimulation function to account for unspecified processes had a negligible impact on predicted lipid concentrations. This was likely due to the larger timescales of dose protocols and simulated data collection times.

The following relationship between CYP450 levels and metabolic clearance was determined to be flexible enough to model the data over a wide range of doses:

(4) $$\begin{array}{c} v_{\mathrm{cl}}=v_{0}\frac{A^{\text{CYP}}}{A_{0}^{\text{CYP}}}, \end{array}$$

where *v*_cl⁡_ is metabolic clearance as a function of CYP450 concentration (mL/h) and *v*_0_ is the basal metabolic clearance under low exposure and negligible induction conditions (mL/h).

The rate of metabolism of each PCB is the product of the PCB concentration in the neutral lipid space of the liver *C*_nlL_(*A*_nlL_/*V*_nlL_) and the *v*_cl⁡_ for the particular PCB:

(5) $$\begin{array}{c} \frac{{dA}_{\text{n}1\text{L}}}{dt}=Q_{\text{n}1\text{L}}\left(C_{\text{n}1\text{a}}-C_{\text{n}1\text{L}}\right)-v_{0}\cdot \frac{A^{\text{CYP}}}{A_{0}^{\text{CYP}}}\cdot C_{\text{n}1\text{L}}, \end{array}$$

(6) $$\begin{array}{c} \frac{dA^{\text{CYP}}}{dt}=k_{0}-k_{\text{e}}\times A^{\text{CYP}}+k_{0}FC_{\text{IND}}. \end{array}$$

PCB induction and metabolism are congener specific and are functions of structure and classification [2]. Non-ortho PCBs (“dioxin-like” PCBs with no ortho-substituted chlorines) assume a coplanar position and are strong inducers of CYP1A. Multi-ortho-substituted PCBs cannot become coplanar and interact primarily with CYP2B. Mono-ortho PCBs can assume both planar and coplanar positions and are considered “mixed-type” inducers [2, 25, 26]. PCB congeners 138, 153, 170, and 180 are di-ortho; 187 is tri-ortho; and 118 is mono-ortho. In the current dataset, PCB 118 shows significantly higher clearance than other congeners at the high-dose level [11].

To model the difference in PCB 118 metabolism at the high doses, the PBTK model assumes the multi-ortho PCBs are metabolized through the CYP2B pathway, while PCB 118 is metabolized via CYP1A. It was also assumed that both types of PCBs induce CYP2B, but induction of CYP1A by multi-ortho PCBs was negligible. These assumptions were based on *in vitro* studies of different classes of PCBs. A study in rat hepatocytes found that mono-ortho PCBs are primarily metabolized by CYP1A and primarily induce CYP1A (with CYP2B being induced to a lesser extent) [27]. CYP1A induction by PCB 118 has also been shown to be orders of magnitude greater than induction by multi-ortho PCBs [28]. Meanwhile, CYP2B induction from both PCB 118 and multi-ortho PCBs was the same order of magnitude [25]. 

Equations (2) through (6) were applied with parameters to describe both CYP1A and CYP2B kinetics. For each PCB, (5) was applied using a basal metabolic clearance (*v*_0_) specific to that PCB, with the induction scaling factor dependent on the PCB classification (mono-ortho or multi-ortho). Induced clearance of PCB 118 was dependent on the CYP1A ratio, while induction of the others was dependent on CYP2B ratio. For the induction of CYP1A, *C*_IND_ in (6) was assumed to be the concentration of PCB 118 in the neutral lipid space of the liver. For CYP2B induction, *C*_IND_ was assumed to be the total PCB concentration (sum of all 6 PCBs) in the neutral lipid space of the liver. This assumes an additive effect on CYP2B induction, with each PCB having equal weight. Separate values for the induction factor *F* were used to describe CYP1A and CYP2B induction.

Parameters for baseline CYP450 dynamics were obtained from literature and are summarized in Table 3. For simplicity, the same parameters were used for both CYP1A and CYP2B enzymes and were obtained from a model for CYP1A [17]. Alternatively, CYP2B-specific parameters from a similar PBTK-induction model can be used [22]. A recent study in female rats estimated CYP1A content to be a factor of two greater than CYP2B in the liver [29], which could also be used to inform the model. It was found that optimizing the model using separate 1A/2B parameters produced nearly identical final results, due to the fact that the synthesis and degradation parameters were proportionally the same. Additionally, the initial amounts and the synthesis/degradation rates are not entirely identifiable and are related via the steady-state mass balance. Since the induction in this model will only be affected by the increase of CYP450 relative to the baseline, the actual baseline values are less important, and some adjustments can be made to those assumptions without reoptimization of induction factor *F*.

### 2.4. Hierarchical Bayesian Model

**Figure 1:**
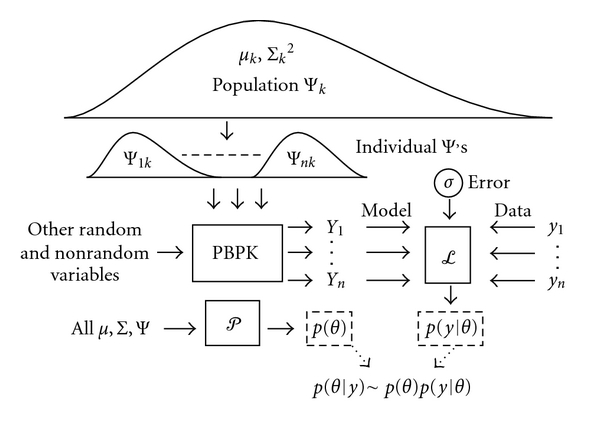
Schematic of a hierarchical Bayesian framework (adapted from [19]).

Interanimal variation existed in the data which could not be attributed to differences in physiology or tissue lipid content alone. It was assumed that this inter-rat variation can be attributed to basal metabolic clearance *v*_0_. A hierarchical model for basal clearance was constructed to optimize the population distribution of *v*_0_ to the observed data (Figure 1).

 A generalized population model assumes random variable Ψ_*ik*_ (where *i* denotes an individual within the population, and *k* denotes the particular variable) is derived from a distribution of mean *μ*_*k*_ and standard deviation Σ_*k*_. Both random and nonrandom variables are used as inputs to the PBTK model to predict *Y*_*i*_. The likelihood function *L* calculates the probability that *Y*_*i*_ is an adequate prediction of data *y*_*i*_ given the set of random variables. The prior function *P* calculates the probability of all random variable values conditional on their population assumptions. The posterior probability is proportional to the product of the likelihood and prior.

A lognormal error function was implemented as the likelihood, which assumes the log of data measurements *y*_*i*_ are scattered in a normal distribution from the log of their corresponding model predictions *Y*_*i*_:

(7) $$\begin{array}{c} \log y_{i}=\log Y_{i}+N_{\text{rnd}}\left(\mu =0,\sigma \right). \end{array}$$

The population and error parameters to be estimated are summarized in Table 4. It was assumed that metabolic clearances at the individual level (*v*_0_) were derived from a lognormal population distribution defined by *μv*_0_ and Σ*v*_0_. Priors on *μv*_0_ for each PCB were set as wide and noninformative. The priors for population variances Σ*v*_0_ for each PCB were assumed to be inverse-gamma with a shape parameter of 1 (indicating large uncertainty), and a scale parameter of 0.8 (the initial assumption on the lognormal Σ). The prior probabilities of individual-level parameters were calculated using the values of population *μ* and Σ for each parameter at the current iteration. Upper and lower limits for the uniform priors on mean population basal clearances (*μv*_0_) were set by observing model behavior at extreme values. It was determined that scaling the basal rate by body-weight^0.75^ slightly improved convergence and model fit, due to the increasing body weight over the 90-day period, and variation in body weight of the studied population. Non-informative distributions were used for the priors on *σ*. Since three tissues were measured (plasma, liver, and fat), three separate values for *σ* were optimized.

During the testing phase of the optimization, interindividual variation in cardiac output, fractional organ volumes, and blood flow rates were incorporated by using informative population priors based on standard literature values for Sprague-Dawley rats. This involved optimizing individual-level physiology while holding population-level distributions constant. It was found that nearly identical final results were obtained regardless of whether variation in physiology was incorporated into the model. In order to improve convergence, mean values for organ physiology were used. Individual-level data of measured liver weight ratios and the change in body weights over the course of the study were still incorporated into the PBTK model during the optimization.

### 2.5. Computational Implementation

The PBTK model was developed in the MATLAB software environment [30], while implementing an open-source Metropolis-Hastings toolbox [31]. The PBTK model is available from the institute website (http://www.ccl.rutgers.edu/onlineCodes/PCBmixturePBPK) and is also provided as a supplemental file. Markov-Chain Monte Carlo (MCMC) with Metropolis sampling was used to iteratively converge to the posterior distribution. The number of PBTK model parameters for each of the 142 rats, added with population and error parameters, leads to over 1000 parameters in total. Convergence issues arise with such high dimensions and noninformative priors. Additionally, each Metropolis step requires the solution of over 800 systems of differential equations, and multiple independent Markov chains are required to assess convergence. Since the system might not converge for 100,000 iterations, it was necessary to apply simplifying assumptions to reduce model evaluations and improve convergence.

Because a minimal induction effect was observed at the lowest dose [11], the parameter optimization was decomposed into two steps. For the first step, induction was neglected and the model was optimized using only the low-dose data in order to obtain the basal metabolic rate (*v*_0_) for each PCB. The resulting population distributions were then used as informative priors in the second step. For step 2, induction was incorporated in the model, and parameters were optimized using only data for the two high doses. While MCMC was still performed on the individual-level values for *v*_0_ in step 2, they were defined by stronger population priors than in step 1. The population mean and variance for *v*_0_ were not updated in step 2, since the final distribution of all individual-level clearances from step 2 remained consistent with the population priors. Had any anomalies been identified (i.e., many individual-level parameters being optimized at the upper or lower limits), the population parameters would have been reoptimized in the second step. The model/data error parameters (*σ*) were optimized in both steps, since it was observed that allowing these parameters to freely explore the space improved convergence.

Splitting the problem into two steps helped to reduce convergence problems, since basal and induced metabolic clearances are inherently nonidentifiable. Interindividual variation of the induction factor *F* was neglected in step 2 (i.e., the prior probability on Σ*F* was assumed to be extremely small). Assuming negligible variation on *F* eliminates the need to estimate individual-level values for each rat and can prevent poor mixing of the Markov chains. Interindividual variation in metabolism would be accounted for by variations in basal clearance. Two induction factors (one each for 1B and 2A induction) would be optimized to fit the entire population.

For both step 1 and step 2 of the parameter optimization, three sets of independent Markov chains were initiated using over dispersed initial guesses. After adjustments in the random-walk parameters to optimize the acceptance rate (it was determined that the optimal acceptance rate for this system was approximately 10% [32]), the chains were run for 50,000 iterations. The chains were considered converged if the Gelman-Rubin convergence statistic was close to 1 for the parameters from all three independent sets of chains [33]. The PBTK models and Metropolis sampler were implemented in MATLAB on a cluster of multi core processors. Convergence of the Markov chains typically occurred after 80,000 iterations and three days computational time.

## 3. Results and Discussion

### 3.1. Posterior Distributions

Results are summarized in Table 4. Mean basal clearances ranged between 0.017 and 0.038 mL/h/kg^0.75^. The population lognormal standard deviations for basal clearances (Σ*v*_0_) were reduced by over half for most of the PCBs, and metabolic clearance was predicted to deviate from the mean by a factor of 2 in the population. Congener 187 was the only PCB to have a lognormal standard deviation greater than 0.5 for basal clearance. PCB 153 clearance had exhibited poor convergence, as indicated by the Gelman-Rubin statistic, despite repeated optimization attempts. The mono-ortho congener 118 had the highest basal clearance, which may be due to a slight induction effect at the lowest dose. Since distributions for basal metabolic rate of all 6 PCBs were relatively similar, an additional MCMC analysis was performed for step 1 assuming a single population distribution for all PCB clearances *v*_0_^all^. The population distribution of *v*_0_^all^ was in agreement with those determined for the individual PCBs and represents a condensed posterior distribution for all six PCBs. Additionally, better convergence was achieved for the lumped standard deviation. Step 2 of the MCMC analysis (determination of induction parameters) was performed using the 6 separate PCB distributions.

### 3.2. Model Evaluation

**Figure 2:**
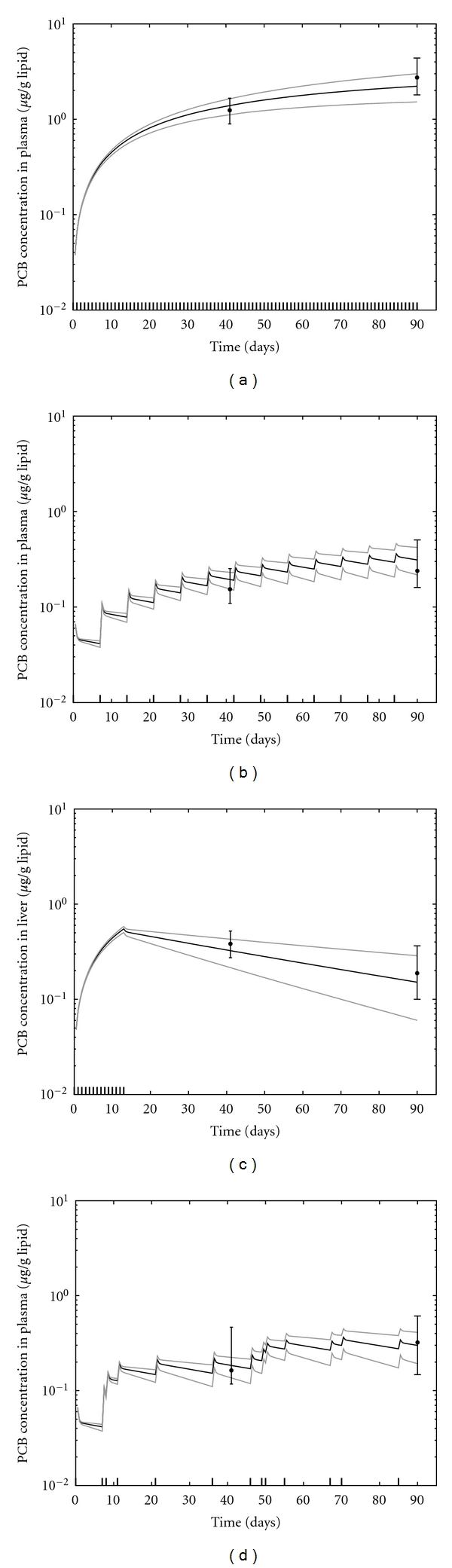
Model predictions and data for all PCB concentrations in tissue lipids at the 5 *μ*g/kg dose level, and the four dose protocols ((a) Daily, (b) weekly, (c) daily to day 13, (d) nonperiodic). Data points represent the median of all PCBs for all rats at time of sacrifice, and error bars represent the minimum and maximum measured values. Modeled results represent the median and 95% confidence interval of 1000 model runs using parameters randomly sampled from the posterior distributions.

**Figure 3:**
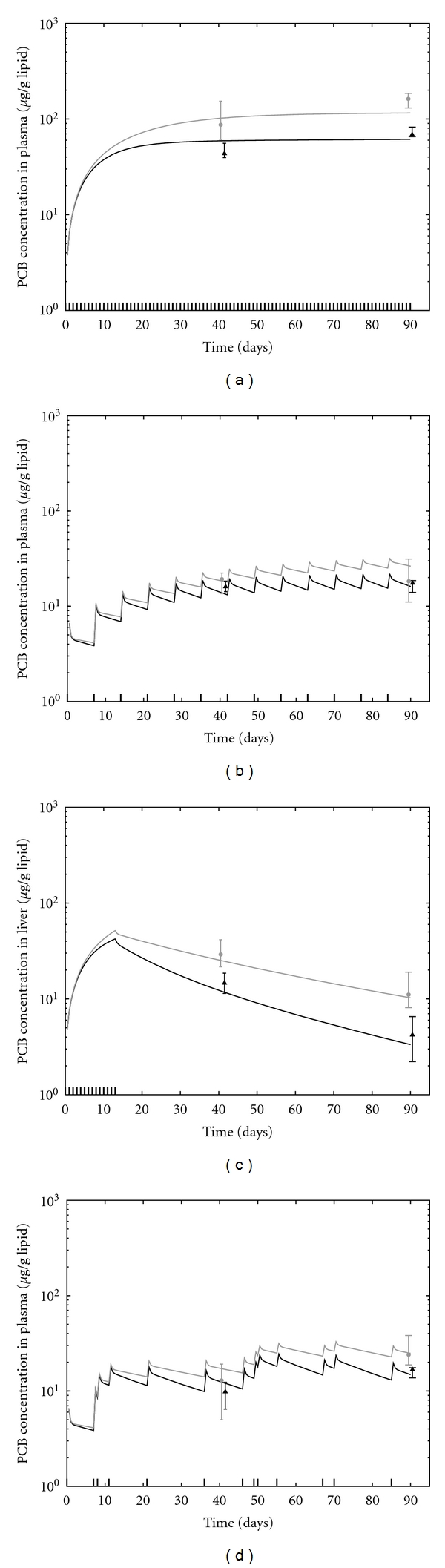
Model predictions and data for all PCBs at the 500 *μ*g/kg dose level and the four dose protocols. Data represents the median measurements across rats at time of sacrifice (grey circle: all multi-ortho PCBs, black triangle: PCB 118). Modeled results are for the median over all multi-ortho PCBs (grey line), and the median results for PCB 118 (black line), resulting from 1000 model runs using parameters randomly sampled from the posterior distributions.

**Figure 4:**
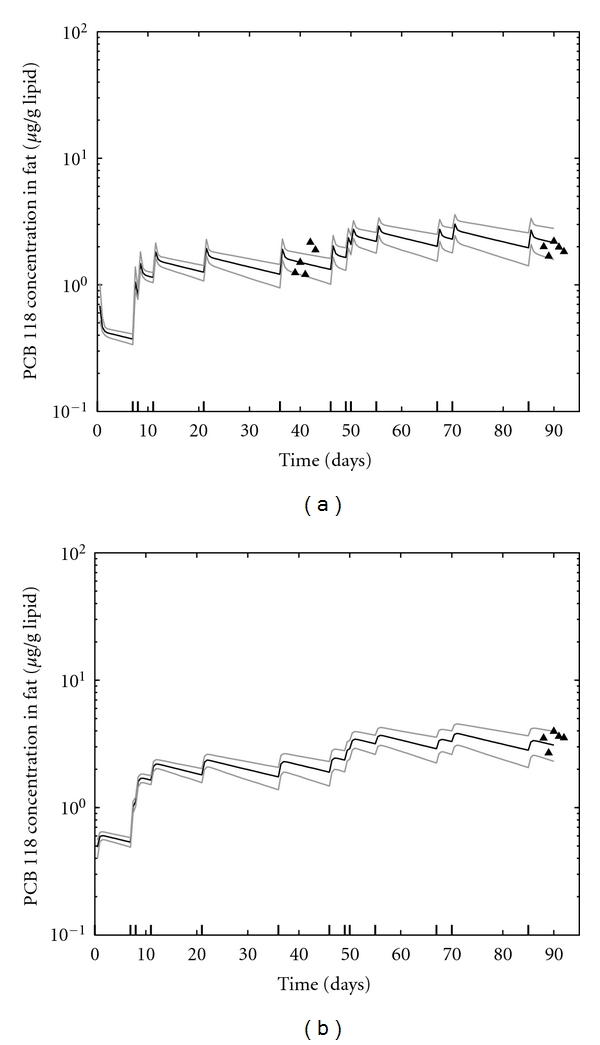
Model predictions for PCB 118 in liver (a) and fat lipids (b) for the nonperiodic dose scenario at the 50 *μ*g/kg dose level. Data for all rats are shown (black triangles), and measurements for each rat at time of sacrifice have been shifted slightly on the *x*-axis. Modeled results are the median and 95% confidence interval of 1000 model runs using parameters randomly sampled from the posterior distribution.

Monte Carlo simulations consisting of 1000 model runs using parameters randomly sampled from the posterior distributions (Table 5) were performed to assess the behavior of the population model. For these simulations, median population *μ* and Σ were used to randomly generate individual-level clearance parameters so that the effect of parameter variability could be observed.  Figure 2 illustrates the variation of the population model and experimental data at the 5 *μ*g/kg dose level. At this low dose level, data and model predictions for both classes of PCBs (multi-ortho and mono-ortho) are similar across dose protocols and tissue type. The variation in model outputs as a function of parameter variability was in agreement with the amount of scatter observed in the data. At the highest dose level, metabolic clearance differs between PCB 118 and the multi-ortho PCBs (Figure 3). The magnitude of this difference was also a function of dose protocol. The model was also able to capture differences in the time profiles between tissues that were due to lipid content (Figure 4).

**Figure 5:**
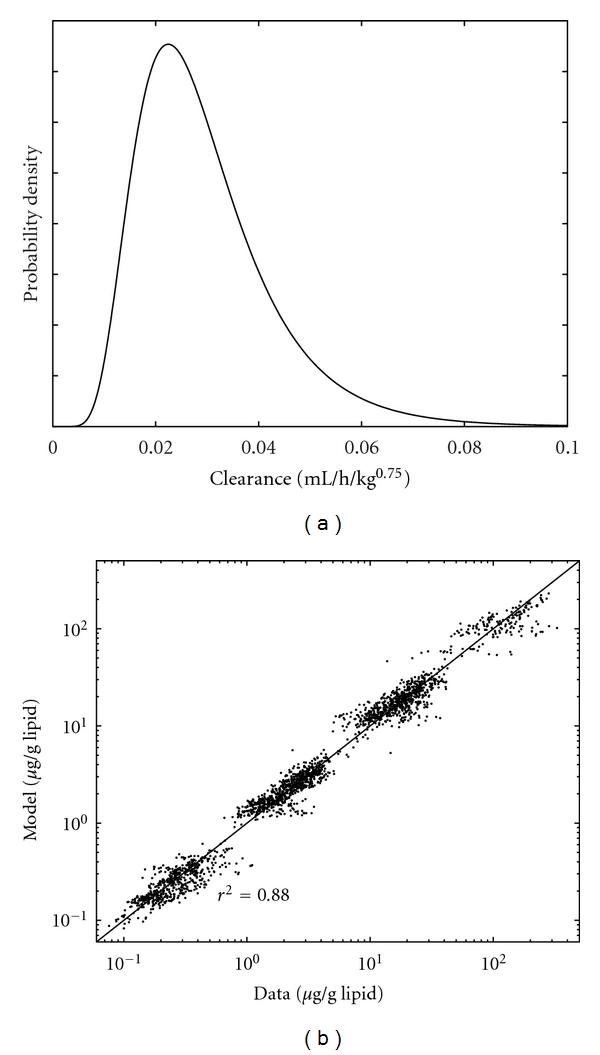
Population distribution of consolidated basal metabolic clearance *v*_0_^all^ (a), and scatter plot of PBTK model predictions versus measured data using the optimized individual-level values of the Markov chains (b).

The performance of the induction model remained consistent with previous observations by Emond et al. [11]. At the highest continuous dose level, metabolic clearance of the multi-ortho PCBs may increase by a factor of 3, while clearance of PCB 118 may increase by a factor of 5. Increases in metabolic rate varied by dose protocol, due to the dynamic behavior of the CYP450 balance. The induction effect is the greatest, and the difference between PCB 118 and multi-ortho PCB concentrations is the largest, when doses occur daily as opposed to sporadically (Figure 3). At the lowest dose, the induction model predicts negligible increase in metabolic clearance for both PCB groups. The model was able to simulate induction as a consistent and continuous function across all dose levels and protocols, while reproducing observed data. A scatter plot comparing all data with model results (using the individual-level posterior values sampled from the converged Markov chains) is presented in Figure 5, along with the condensed posterior distribution of basal metabolic clearance of all PCBs.

 The lipid-based toxicokinetic model does have inherent limitations. Because these models do not include partition coefficients for each PCB/tissue combination, they cannot capture differences in the ordering of PCB concentrations that are observed between different tissues of the same rat. While PCB 118 is observed as having the lowest concentration in all tissue lipids for most of the rats, there is a slight tissue dependency among the ordering of multi-ortho PCBs. For example, PCB 180 was usually observed to have the second-highest PCB concentration in fat lipid but had either the lowest or second-lowest concentration in plasma and liver lipids. PCB 187 usually had the highest concentrations in plasma and fat, but not liver. The magnitude of the differences between multi-ortho PCB concentrations was relatively small. However, the effect is somewhat visible in the model/data scatter plot (Figure 5(b)), where trends exist in each cluster due to a consistent over- or underprediction of specific PCB-tissue combinations. Slight correlations between the clearance parameters are also inherent in the model, and an attempt was made to use the multivariate prior distribution from step 1 in the step 2 optimization. However, convergence issues and the lack of congener-specific tissue affinity parameters ultimately made an accurate characterization of these correlations infeasible.

The other modeling simplification involves the estimation of a basal metabolic rate based on the low-dose data. If the metabolic rate is significantly increased at low exposures due to induction, optimizations at the higher doses will be biased. For low doses of PCB 126 (a potent dioxin-like PCB) in rats, *in vivo* studies have shown significant increases in EROD (7-ethoxyresorufin-O-deethylase) activity (which is indicative of CYP1A). A 10-fold increase in EROD activity has been observed after a single 7.5 *μ*g/kg dose [7], and a 95-fold increase was observed for 1 *μ*g/kg/day exposure [34]. Low-dose induction of CYP2B, indicated by PROD (7-pentoxyresorufin-O-dealkylase) activity, has been observed due to mixtures of mono-ortho and multi-ortho PCBs [25]. Rats orally exposed to PCB 153 (one of the congeners in the current study) showed a 4-fold induction of CYP1A and a 20-fold induction of CYP2B at 3 mg/kg [35]. Since the current work concerns doses at the *μ*g/kg level of the relatively low potent PCBs, the CYP induction implemented here (maximum of about 5-fold) does not contradict earlier studies. Additionally, since the increase in metabolic rate between the 5 and 50 *μ*g/kg dose levels was very small in this study, the assumption of negligible rate increase between the “true” basal rate and the rate estimated at low-dose appears to be rational.

Other simplifying assumptions include linearization of the biological exposure response, neglecting Ah-receptor binding, and discretizing the induction model into 2 PCB groups (multi-ortho and non-ortho) and 2 enzyme groups (CYP1A and CYP2B). Competitive inhibition for P450s [5], regional hepatic CYP450 induction [6], and induction of Phase-II metabolic enzymes [7] were also neglected. Such model complexities lie outside the scope of this work and would have made MCMC analysis infeasible due to weak prior information and nonidentifiable parameters. While the Bayesian framework implicitly incorporated these and other discrepancies into the model/data error for each tissue, the actual model/data error can never be truly known. It is typically assumed that the collection of additional replicates will reduce the uncertainties. However, there is a point where additional replication will not yield model improvements. An observation to this effect occurred during an initial testing phase of the Bayesian framework. The model optimization results were originally evaluated by a “data-splitting” technique, where one rat from each dose-level/dose-protocol/sacrifice-time was omitted from the optimization dataset. The optimized model was then tested against this omitted data to assess performance. It was later found that optimizing the model to the full data set produced nearly identical posterior values (including model/data error) as optimizing to the dataset containing approximately 17% fewer rodents. Future studies involving mixtures of contaminants having very similar toxicokinetic properties would benefit from a value-of-information analysis at the experimental design phase, in order to reduce the number of test rodents needed to develop a mixture model.

## 4. Conclusions

This is the first application of a large-scale population Bayesian analysis to a mixture PBTK model. Despite the lack of partition coefficients and reduced degrees of freedom, the optimized model was capable of reproducing experimental data in multiple tissue lipids for a wide range of PCB dose levels and protocols. The application of a linear induction dose-response model, and the use of lipid-based concentrations, illustrated parsimonious alternatives to highly complex nonlinear models containing large numbers of parameters. While the current modeling effort sought to avoid the issue of nonidentifiability or overparameterization, further improvement could be made by incorporation of a fully mechanistic model for CYP450 induction. However, such a model would likely require predictions of PCB concentrations outside of the lipid space, and the type of additional data needed would depend on the aims and scope of the proposed mechanistic model.

## Figures and Tables

**Table 1:** Lipid content of rat tissues [11, 14].

Tissue	NLE^†^	NLE/total lipid
Blood	0.0019	0.576
Plasma	0.0009	0.748
Fat	0.8536	0.998
Liver	0.0425	0.710
Rapidly perfused	0.0425	0.710
Poorly perfused	0.0120	0.632

^†^Neutral lipid equivalent ratio (mL NLE/mL tissue).

**Table 2:** Physiological values for a standard 225 g rat (adapted from [11]).

Tissue	Conventional model^†^	NLE-based model
Blood flow rates		

Fat	448 mL/h	0.85 mL lipid/h
Liver	1245 mL/h	2.36 mL lipid/h
Rapidly perfused	2540 mL/h	4.82 mL lipid/h
Poorly perfused	747 mL/h	1.42 mL lipid/h
Cardiac output	4980 mL/h	9.45 mL lipid/h

Volumes		

Blood	20.0 mL	0.038 mL lipid
Fat	17.5 mL	14.938 mL lipid
Liver	10.0 mL	0.425 mL lipid
Rapidly perfused	12.5 mL	0.531 mL lipid
Poorly perfused	167.5 mL	2.010 mL lipid

^†^Physiological parameter values obtained from [15, 16].

**Table 3:** Basal CYP1A/2B parameters [17].

Parameter	Symbol	Units	Value
Initial steady-state CYP1A/2B	*A*_0_	nmol/g protein	0.1**^†^**
CYP1A/2B degradation	*k*_*e*_	h^−1^	0.04
Basal CYP1A/2B production	*k*_0_	nmol/h/g protein	0.004

**^†^**Computed from the steady-state mass balance.

**Table 4:** Population-level parameters to be estimated by Bayesian analysis.

Parameter	Unknowns	Prior
Basal metabolic rate^†^	*μv*_0_	Wide uniform
	Σ*v*_0_	Inverse gamma
Induction factor	*F* ^2B^	Wide uniform
	*F* ^1A^	Wide uniform
Model/data error^‡^	*σ*	Wide uniform

^†^One each for PCBs 118, 138, 153, 170, 180, 187, in (mL/h/kg^0.75^).

^‡^One each for plasma, liver, and fat.

**Table 5:** Population posterior distributions.

Parameter*	*μ* (CV/R)^†^	Σ (CV/R)
*v*_0_^118^	0.038 (0.02/1.0)	0.24 (0.13/1.2)
*v*_0_^138^	0.026 (0.02/1.2)	0.35 (0.17/1.1)
*v*_0_^153^	0.025 (0.02/1.3)	0.35 (0.16/1.5)
*v*_0_^170^	0.029 (0.02/1.0)	0.38 (0.16/1.0)
*v*_0_^180^	0.035 (0.02/1.1)	0.31 (0.15/1.0)
*v*_0_^187^	0.017 (0.02/1.1)	0.55 (0.25/1.0)
*v*_0_^all^	0.027 (0.03/1.0)	0.43 (0.08/1.1)

*F* ^2B^	0.0025 (0.01/1.0)	
*F* ^1A^	0.045 (0.02/1.2)	

*σ*_fat_	0.30 (0.06/1.0)^‡^ 0.23 (0.04/1.0)	
*σ*_plasma_	0.19 (0.04/1.2) 0.23 (0.03/1.3)	
*σ*_liver_	0.40 (0.05/1.1) 0.36 (0.03/1.0)	

*Units for *v*_0_ are (mL/h/kg^0.75^); *F* is unitless.

^†^CV: coefficient of variance of chain, R: Gelman-Rubin.

^‡^One value each for step 1 (top) and step 2 (bottom).
